# Ultrasound-detected gastric changes related to zolbetuximab-induced emesis: a case series

**DOI:** 10.3389/fonc.2025.1686245

**Published:** 2025-11-28

**Authors:** Kazuaki Harada, Koichi Ishida, Shiho Kaneko, Tatsuya Yokoyama, Shintaro Sawaguchi, Takeaki Nakamura, Yasuyuki Kawamoto, Satoshi Yuki, Mutsumi Nishida, Naoya Sakamoto, Yoshito Komatsu

**Affiliations:** 1 Division of Cancer Center, Hokkaido University Hospital, Sapporo, Japan; 2 Department of Gastroenterology and Hepatology, Hokkaido University Hospital, Sapporo, Japan; 3 Department of Gastroenterology, Japanese Red Cross Kitami Hospital, Kitami, Japan; 4 Department of Management Strategy, Hokkaido University Hospital, Sapporo, Japan

**Keywords:** chemotherapy, zolbetuximab, ultrasound imaging, gastric cancer, vomiting, case report

## Abstract

**Background:**

Zolbetuximab prolongs survival in patients with claudin18.2 (CLDN18.2)-positive gastric cancer (GC) and gastroesophageal junction cancer. However, it is associated with an increased risk of emesis, and its underlying mechanism remains unclear.

**Case Presentation:**

This report presents three cases of advanced GC that underwent abdominal ultrasonography (US) during zolbetuximab administration. In two patients with primary gastric lesions, severe nausea occurred during the first treatment cycle. US findings revealed increased echogenicity of the submucosal layer, enhanced layer stratification, and increased blood flow signals, which improved as the emesis subsided. During the second cycle, emesis was milder, and US findings revealed only slight changes. In the third patient who underwent total gastrectomy, no nausea or abnormal US findings were observed.

**Conclusion:**

These US findings indicate gastric tumor tissue injury due to zolbetuximab, which is associated with emesis. US is a valuable tool for further elucidating the mechanisms underlying zolbetuximab-induced emesis and may also contribute to the prediction and prevention of this adverse event.

**Statement:**

Zolbetuximab treatment has been reported to improve survival in patients with HER2-negative, CLDN18.2-positive advanced, unresectable, or recurrent gastric and gastroesophageal junction cancers. However, it is associated with a high incidence of nausea, particularly during the first infusion, and the underlying mechanism remains unclear. We present three cases of advanced or recurrent gastric cancer treated with zolbetuximab, during which abdominal ultrasonography (US) was performed. In two cases with primary gastric tumors, US revealed characteristic changes in the gastric wall at the onset of nausea, suggestive of edema and inflammation. These changes appeared prior to the symptoms and resolved as nausea improved. Nausea during the second infusion was milder, with less pronounced US changes. The third case includes a patient who had undergone total gastrectomy; the patient did not experience nausea, and no gastrointestinal changes were observed on US. This is the first report to describe US findings associated with zolbetuximab-induced nausea. These observations suggest that gastric wall changes detectable using US are closely linked to nausea and may reflect underlying gastric injury. This report also highlights the potential of bedside US monitoring to help predict and prevent nausea during zolbetuximab therapy.

## Introduction

Gastric cancer (GC), including gastroesophageal junction cancer (GEJC), is the fifth most common malignancy and the fifth leading cause of cancer-related mortality worldwide (1). Despite advances in treatment, the 5-year survival rate for patients with GC/GEJC remains <30%, particularly those with advanced, unresectable, or recurrent GC/GEJC (2).

Therapeutic strategies tailored to specific molecular subtypes of the disease have been developed to prolong the survival of patients with unresectable or recurrent GC/GEJC. Trastuzumab with fluoropyrimidine- and platinum-based chemotherapy is the standard first-line treatment for human epidermal growth factor receptor 2 (HER2)-positive tumors (3). Furthermore, the combination of pembrolizumab, an anti-programmed death-1 (PD-1) antibody, with this regimen has shown a survival benefit in patients with HER2-positive tumors with a high programmed cell death ligand 1 (PD-L1) combined positive score (4). The efficacy of anti-PD-1 antibodies, such as nivolumab and pembrolizumab, combined with platinum-fluoropyrimidine chemotherapy in HER2-negative tumors is influenced by PD-L1 expression (5–7). Notably, anti-PD-1 antibodies provide significant survival benefits in patients with microsatellite instability-high tumors, a subset of GC/GEJC with a distinct molecular profile (5, 7).

Claudin18.2 (CLDN18.2), a tight junction protein highly expressed in the normal gastric mucosa and retained in certain GC/GEJC cells, has emerged as a promising therapeutic target (8). Zolbetuximab, a first-in-class chimeric monoclonal antibody that targets CLDN18.2, exerts antitumor effects through antibody-dependent cellular and complement-dependent cytotoxicity (9, 10). Building on promising results in early-phase clinical trials (11, 12), two global phase III trials were conducted to evaluate the efficacy and safety of zolbetuximab in patients with CLDN18.2-positive GC/GEJC. The SPOTLIGHT trial demonstrated that zolbetuximab combined with mFOLFOX6 significantly improved progression-free survival (PFS) and overall survival (OS) compared with placebo (13). Similarly, the phase III GLOW trial demonstrated significant improvements in PFS and OS with zolbetuximab plus CAPOX compared with CAPOX alone (14). Based on these findings, zolbetuximab was approved in Japan as a treatment option for patients with HER2-negative, CLDN18.2-positive advanced, unresectable, or recurrent GC/GEJC in March 2024.

Despite its survival benefits, zolbetuximab is associated with a higher incidence of emesis (13, 14). Emesis that occurs immediately after zolbetuximab administration is transient, improves upon treatment discontinuation, and is attenuated with subsequent doses. However, the mechanism underlying the onset of emesis remains poorly understood. Furthermore, no effective treatment or preventive strategy has been established for zolbetuximab-induced emesis, highlighting the need for further investigation into its underlying mechanisms. for zolbetuximab-induced emesis. In this case series, we observed characteristic findings associated with zolbetuximab-induced emesis in patients with advanced or recurrent gastric cancer. Abdominal ultrasonography (US) was performed during zolbetuximab administration using a pocket-sized, wireless ultrasound device (Vscan Air CL; GE HealthCare, Chicago, IL, USA). Our findings may contribute to a better understanding of the mechanism of zolbetuximab-induced emesis and the development of preventive strategies.

### 1.1 Case 1

A 71-year-old woman was referred to our department with a diagnosis of GC with peritoneal metastasis (T4aN2M1 cStage IV). The tumor infiltration caused stenosis throughout the stomach, accompanied by passage obstruction. The pathological diagnosis was signet-ring cell carcinoma. Immunohistochemistry (IHC) revealed CLDN18 positivity (≥75% of tumor cells) and HER2 negativity (IHC score: 1+). Based on these findings, systemic chemotherapy with mFOLFOX6 (fluorouracil, leucovorin, and oxaliplatin) plus zolbetuximab was initiated.

**Figure 1:**
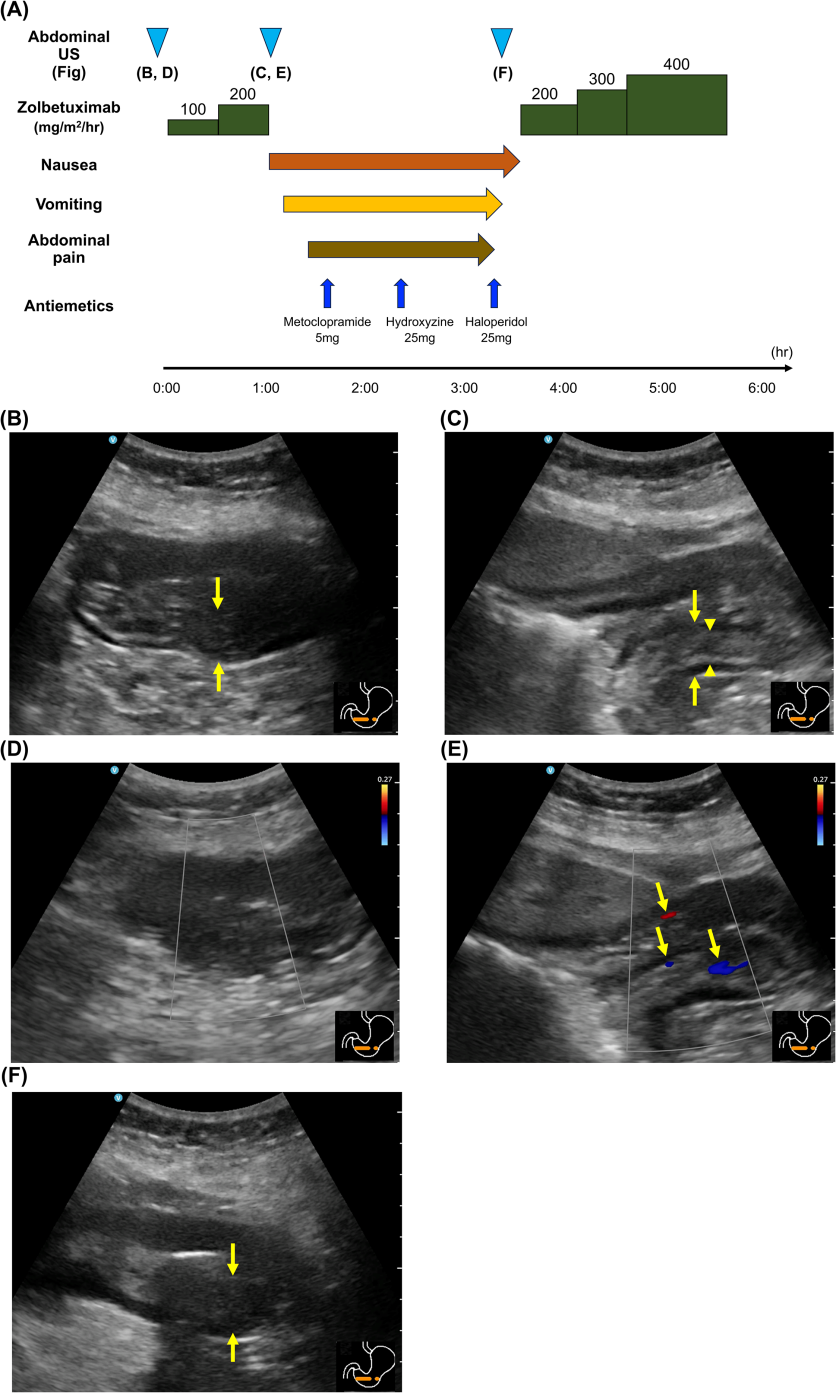
**(A)** Treatment course with the first cycle of zolbetuximab in Case 1. **(B)** Baseline ultrasound (US) showed the primary lesion of gastric cancer as a thickened hypoechoic wall with an indistinct layer structure (arrows). **(C)** At the onset of nausea, increased echogenicity of the submucosal layer (arrowheads) and enhanced layer stratification (arrows) were observed. **(D)** Color Doppler imaging at baseline revealed no detectable blood flow signals in the primary lesion. **(E)** At the onset of nausea, an increase in blood flow signals was observed (arrows). **(F)** With improvement in nausea, the echogenicity of the submucosal layer decreased, and the layer structure became indistinct again (arrows).

**Figure 1A** shows the clinical course during the first zolbetuximab administration. After preventive antiemetic therapy, which included olanzapine, palonosetron, dexamethasone, and fosaprepitant, zolbetuximab (800 mg/m^2^) was started at a rate of 100 mg/m^2^/h. Since no nausea was reported, the infusion rate was increased to 200 mg/m^2^/h after 30 min. However, emesis and abdominal pain occurred 35 min after the rate was increased, leading to the discontinuation of zolbetuximab. US at the onset of emesis showed increased echogenicity in the submucosa and clear delineation of the layer structure compared with the preadministration findings (**Figures 1B, C**). Color Doppler imaging showed increased blood flow signals in the submucosal layer (**Figures 1D, E**). Metoclopramide, hydroxyzine, and haloperidol were administered after zolbetuximab discontinuation to manage nausea. The emesis resolved after 3 h, and zolbetuximab administration was resumed. No emesis recurrence was observed after resumption. The increased echogenicity in the submucosa and clear delineation of the layer structure disappeared as the emesis subsided before zolbetuximab administration was resumed (**Figure 1F**).

**Figure 2:**
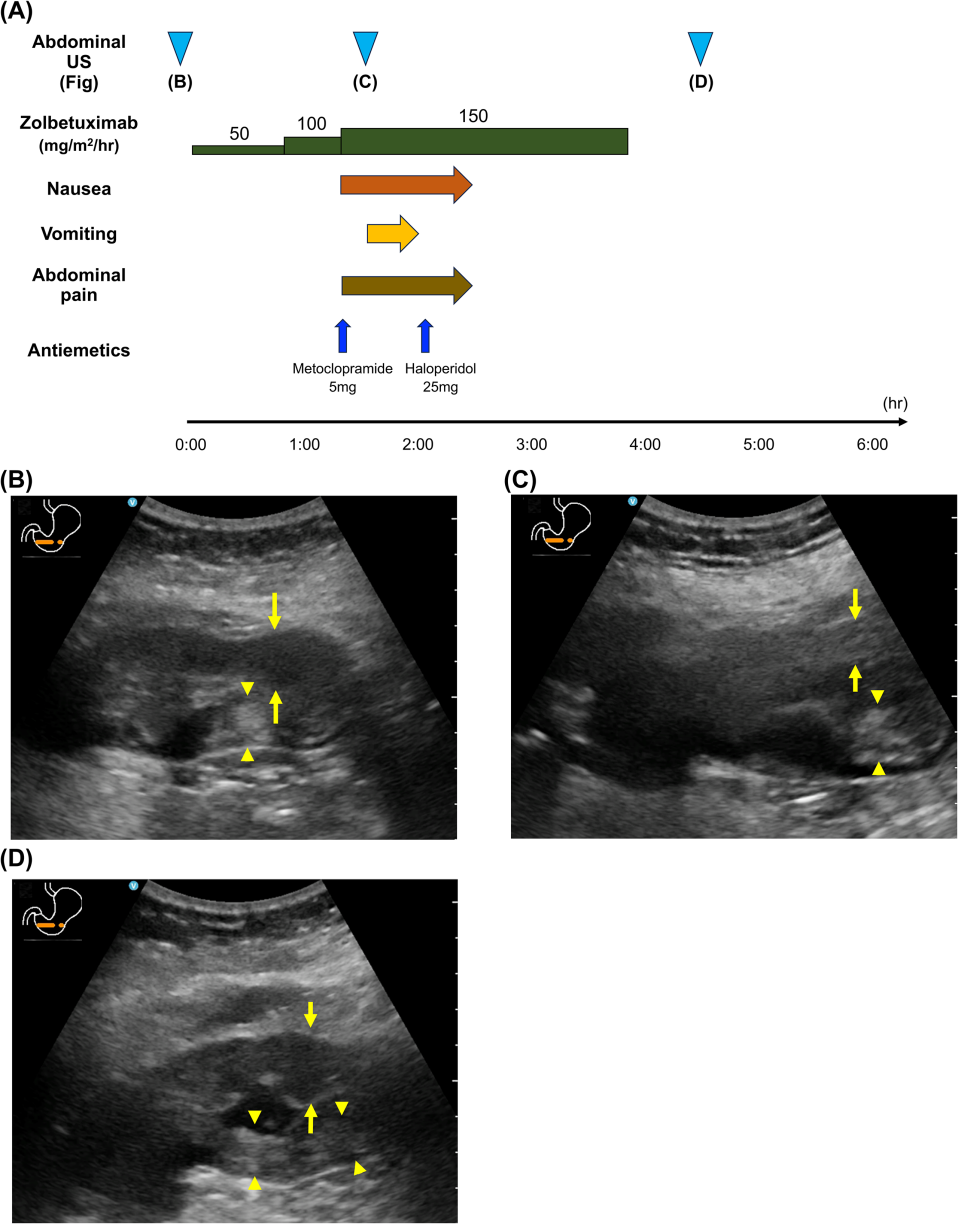
**(A)** Treatment course with the second cycle of zolbetuximab in Case 1. **(B)** At baseline, increased echogenicity was observed in part of the posterior wall (arrowheads), which was not observed before zolbetuximab administration. The anterior wall remained hypoechoic with an indistinct layered structure (arrows), similar to the preadministration findings. **(C)** At the onset of nausea, slightly increased echogenicity in the anterior wall (arrows) and increased echogenicity of the submucosal layer of the posterior wall (arrowheads) with enhanced layer stratification were observed. **(D)** After the completion of zolbetuximab administration, most of the posterior wall exhibited increased echogenicity with indistinct wall layer stratification (arrowheads), whereas the anterior wall structure became indistinct again (arrows).

On day 14, grade 3 neutropenia was observed. Therefore, the second cycle of FOLFOX plus zolbetuximab therapy (400 mg/m^2^) was initiated 21 days after the first cycle. **Figure 2A** shows the clinical course during the second infusion. Zolbetuximab was initiated at an infusion rate of 35 mg/h, and the rate was gradually increased while monitoring for nausea. Emesis occurred, but it was milder than that during the first cycle, and zolbetuximab administration was completed. Before zolbetuximab administration, US revealed partial high echogenicity in the gastric wall and a reduction in gastric wall thickening compared with the findings from the first treatment cycle, indicating a therapeutic response to the initial treatment (**Figure 2B**). At the onset of nausea, a slight increase in echogenicity in the anterior wall and increased echogenicity of the submucosal layer of the posterior wall with enhanced layer stratification were observed (**Figure 2C**). After the completion of zolbetuximab administration, most of the posterior wall exhibited increased echogenicity with indistinct wall layer stratification, whereas the anterior wall structure became indistinct again (**Figure 2D**).

### 1.2 Case 2

A 65-year-old woman was referred with a diagnosis of GC with peritoneal metastasis (T3N0M1 cStage IV). Upper gastrointestinal endoscopy revealed a type 4 tumor extending from the gastric body to the fornix. The pathological diagnosis was signet-ring cell carcinoma. IHC revealed CLDN18 positivity and HER2 negativity (IHC score 0). Consequently, FOLFOX plus zolbetuximab therapy was initiated.

**Figure 3:**
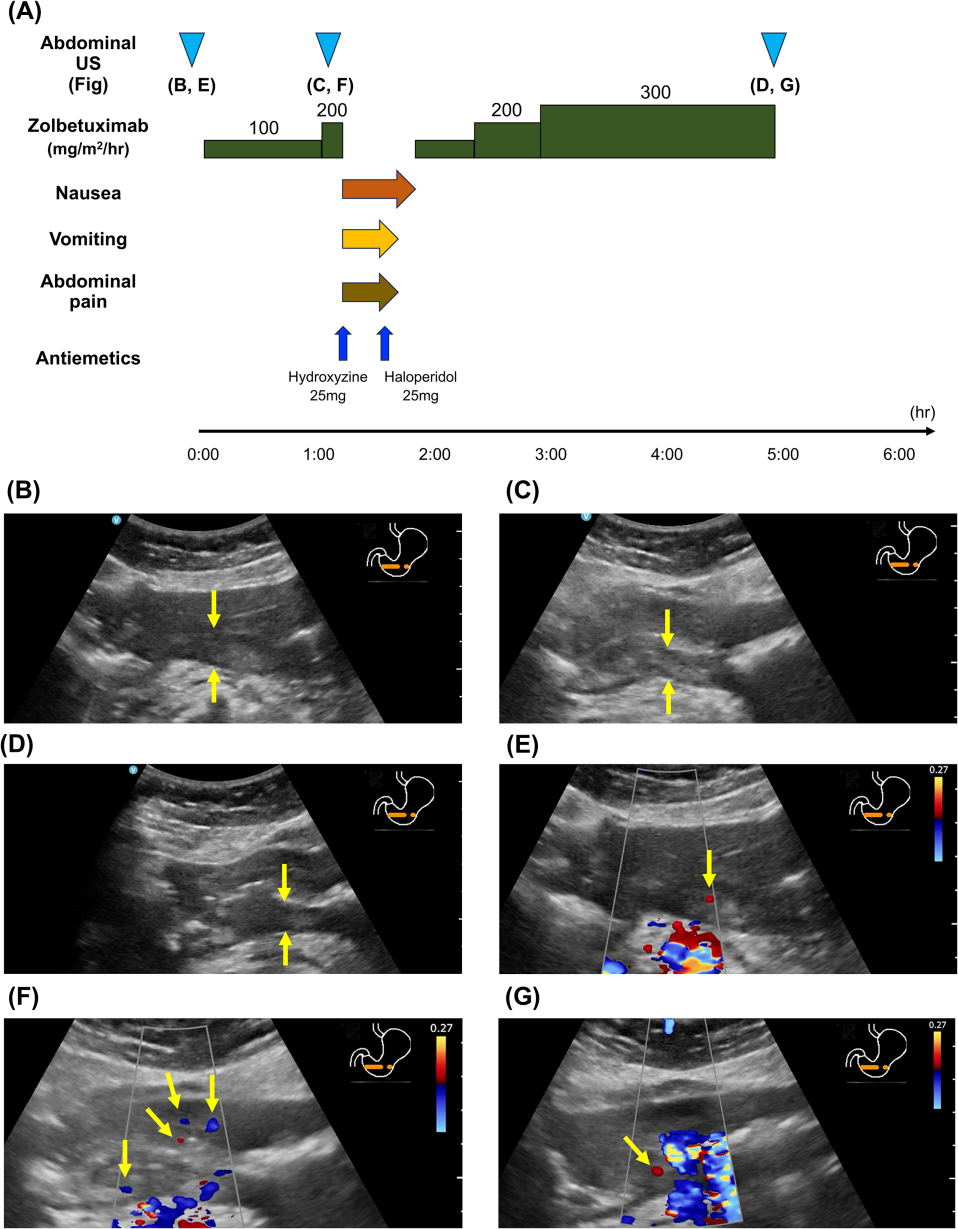
**(A)** Treatment course with the first cycle of zolbetuximab in Case 2. **(B)** At baseline, **(C)** after 1 h of zolbetuximab administration, and **(D)** after the completion of zolbetuximab administration. At the onset of nausea, increased echogenicity of the submucosal layer and enhanced layer structure were observed. Color Doppler US findings **(E)** at baseline, **(F)** at the onset of nausea, and **(G)** after the completion of zolbetuximab administration. An increase in blood flow signals was observed during zolbetuximab administration.

**Figure 3A** shows the clinical course following the first zolbetuximab administration. Following premedication with the same antiemetic agents as in Case 1, zolbetuximab (800 mg/m^2^) was administered at an infusion rate of 100 mg/m²/h. After 1 h, the rate was increased to 200 mg/m²/h. Nausea and vomiting were not observed. However, abdominal US revealed an increase in the echogenicity of the gastric submucosa and a more distinct gastric wall layer structure than before zolbetuximab administration (**Figures 3B, C**).

The patient developed nausea, vomiting, and abdominal pain 10 min after increasing the infusion rate to 200 mg/m²/h, and zolbetuximab administration was discontinued. Hydroxyzine and haloperidol were administered, which alleviated symptoms within 40 min, and zolbetuximab was resumed. No gastrointestinal symptoms were observed, and zolbetuximab administration was completed with gradual dose escalation. Abdominal US findings improved at the end of zolbetuximab administration (**Figure 3D**). A slight increase in blood flow signals was observed during zolbetuximab administration (**Figures 3E–G**).

Grade 3 neutropenia was observed, and the second cycle of FOLFOX plus zolbetuximab therapy was initiated 21 days after the first cycle. After zolbetuximab initiation (400 mg/m^2^), the patient reported abdominal pain, leading to a temporary zolbetuximab discontinuation (**Supplementary Figure 1A**). However, the symptoms were mild, and nausea and vomiting were not observed. US findings revealed decreased wall thickening and slightly elevated echogenicity of the wall compared with the findings before zolbetuximab initiation. Similar to the first administration, US showed slightly increased gastric submucosal echogenicity after zolbetuximab infusion. However, the changes were milder than those observed during the first cycle (**Supplementary Figures 1B–D**).

### 1.3 Case 3

A 77-year-old man was diagnosed with GC (T4aN1M0 Stage III). Total gastrectomy with D2 lymphadenectomy was performed. The pathological diagnosis was poorly differentiated adenocarcinoma. The patient developed severe perianal pain 4 years postoperatively. CT scan revealed thickening of the rectal wall, and rectal biopsy revealed adenocarcinoma similar to primary GC. Therefore, he was diagnosed with peritoneal recurrence of GC. IHC revealed CLDN18 positivity and HER2 negativity (IHC score 0). CapeOX (capecitabine and oxaliplatin) plus zolbetuximab was initiated.

**Supplementary Figure 2** shows the clinical course of the first zolbetuximab administration (800 mg/m^2^). No symptoms, such as nausea or vomiting, were observed during zolbetuximab administration. Abdominal US revealed no significant changes in the anastomotic site after zolbetuximab administration.

## 2 Discussion

To the best of our knowledge, this is the first report to analyze US findings of GC during zolbetuximab administration. A summary of US findings is provided in the **Supplementary Table 1**. Although reports have indicated gastric wall thickening on CT imaging after zolbetuximab administration (15), abdominal US is a noninvasive and real-time assessment method at the bedside and may serve as a more useful tool for monitoring the effects of zolbetuximab on gastric tumors. Furthermore, because abdominal US can capture changes in the layer structure of the stomach that are difficult to identify on CT, it may allow for more detailed observation of the effects of zolbetuximab on gastric tumors. Moreover, in Case 2, ultrasonographic changes in the gastric wall were observed prior to the onset of nausea. This suggests that bedside US monitoring may facilitate early detection of such changes, enabling adjustments to the zolbetuximab infusion rate before the onset of nausea and thereby improving the management of this adverse effect.

The submucosal layer of the stomach, which is rich in vascular beds, appears hyperechoic on US because of reflection and scattering between tissues with different acoustic impedance, such as blood vessels (16). Most diffuse-type GCs have been reported to show gastric wall thickening that preserves wall stratification on US. When GC cells infiltrate the submucosal layer, stromal reaction-induced fibrosis occurs, thereby reducing echogenicity (17). In this report, two cases of primary GC showed increased echogenicity of the submucosal layer, enhanced layer stratification, and increased blood flow signals after zolbetuximab administration. These changes were rapid and reversible. Although the exact mechanism remains unclear, these findings indicate submucosal edema and inflammatory cell infiltration in gastric tumor tissue following zolbetuximab administration. Zolbetuximab binds to CLDN18.2 on GC cells, inducing antibody-dependent cellular cytotoxicity and complement-dependent cytotoxicity CDC (9, 10). Tumor tissue injury causes vascular dilation and increased permeability, causing plasma components to leak into the extravascular space and leading to interstitial edema. This process may increase the number of tissues with different acoustic impedance, thereby increasing the echogenicity of the submucosal layer. Histopathological studies on normal ferret stomach have shown that zolbetuximab administration causes inflammatory cell infiltration into the gastric mucosa and submucosa and tissue damage, particularly in the superficial mucosal layer (18). Although the extent to which these findings can be extrapolated to human GC remains unclear, the increased echogenicity of the submucosal layer observed in our cases may be attributed to the infiltration of numerous inflammatory cells with different acoustic impedance into the cancer-invaded submucosa following zolbetuximab administration, leading to increased ultrasound beam scattering. These changes in US findings coincided with the onset of emesis. Therefore, zolbetuximab-induced nausea may be attributed to direct tissue injury of the primary gastric tumor. Additionally, the absence of emesis and US-detected changes in Case 3 who underwent total gastrectomy support the finding that zolbetuximab-induced emesis originates from gastric tumor tissue injury.

Although abdominal US findings suggest that zolbetuximab may induce localized injury to the gastric wall, the mechanism by which this injury triggers emesis remains unclear. It is well established that the development of chemotherapy-induced nausea and vomiting (CINV) involves multiple neurotransmitters and receptors, including serotonin and substance P. Cytotoxic agents can stimulate enterochromaffin cells in the intestine via the bloodstream or direct mucosal exposure, resulting in serotonin release. The released serotonin activates the chemoreceptor trigger zone and vagal afferents, ultimately stimulating the vomiting center in the medulla (19, 20). Since zolbetuximab is a monoclonal antibody targeting CLDN18.2, it is unlikely to cause emesis through the exact same mechanisms as traditional cytotoxic agents. However, both serotonin and substance P are also upregulated during gastrointestinal inflammation and function as pro-inflammatory mediators (21). Therefore, if inflammatory cells are involved in zolbetuximab-induced gastric wall injury, it is plausible that nausea and vomiting could occur, at least in part, via pathways similar to those seen in acute-phase CINV.

However, as this case series includes only three cases, the evidence is inherently limited. Therefore, these results should be interpreted as exploratory and hypothesis-generating. Further studies with larger cohorts are needed to validate these findings.

In Cases 1 and 2, emesis was milder during the second cycle of zolbetuximab than during the first cycle, and US findings were less pronounced. This may be due to the decrease in the number of CLDN18.2-positive GC cells following the first treatment cycle. In both cases, gastric wall thickening was reduced, and echo levels partially increased on US at the initiation of the second treatment cycle. These findings may reflect the tumor response to the first treatment cycle. Furthermore, reducing the zolbetuximab dose may mitigate gastric tissue injury and alleviate emesis. Currently, the GENTLE-Z study (jRCTs031240347) is evaluating the impact of reduced initial doses of zolbetuximab on nausea incidence, and its results are awaited.

In addition to small number of cases, this report has some limitations. It was based on information obtained as part of routine clinical practice in our institution. In the presented cases, abdominal US was performed before zolbetuximab administration to confirm the absence of gastrointestinal contents that could increase aspiration risk. Subsequent examinations were conducted to investigate the causes of nausea or abdominal pain. Thus, the examinations were not performed according to any predefined protocol specifically designed to assess gastric wall changes. Although all US examinations were performed using a pocket-sized wireless ultrasound device (Vscan Air CL; 2–5 MHz convex probe), the timing and anatomical sites of ultrasonographic evaluation were not standardized. Abdominal US was generally performed at three time points—prior to zolbetuximab administration, at the onset of nausea, and after symptom resolution—yet consistent evaluation across all cases was not ensured. Observations such as “increased echogenicity” and “enhanced of layer strutification” were determined based on visual consensus among multiple gastroenterologists and sonographers, rather than predefined or quantitative criteria. Therefore, further studies with larger sample sizes and standardized protocols are needed to clarify the changes in gastric US findings after zolbetuximab administration. Additionally, the long-term effects of more than three cycles of zolbetuximab remain unclear and require further investigation. However, despite these limitations, the morphological changes in the gastric wall observed in our cases are consistent with the findings from a preclinical animal study (18), strongly supporting the hypothesis that zolbetuximab-induced nausea is attributable to gastric tissue injury.

In conclusion, this report presented three cases of patients with GC who underwent abdominal US during zolbetuximab administration. The findings indicate that gastric tissue injury caused by zolbetuximab is a key contributor to emesis. Further studies involving larger patient cohorts and using US are needed to further elucidate the mechanisms underlying zolbetuximab-induced emesis and establish effective antiemetic strategies.

## Data Availability

The original contributions presented in the study are included in the article/**Supplementary Material**. Further inquiries can be directed to the corresponding author/s.
