# Supplementary material for: Ultrasound-detected gastric changes related to zolbetuximab-induced emesis: a case series
Source: Front Oncol. 2025 Nov 28;15:1686245. doi: 10.3389/fonc.2025.1686245 (PMC12699232; doi:10.3389/fonc.2025.1686245)

Supplementary Material

##
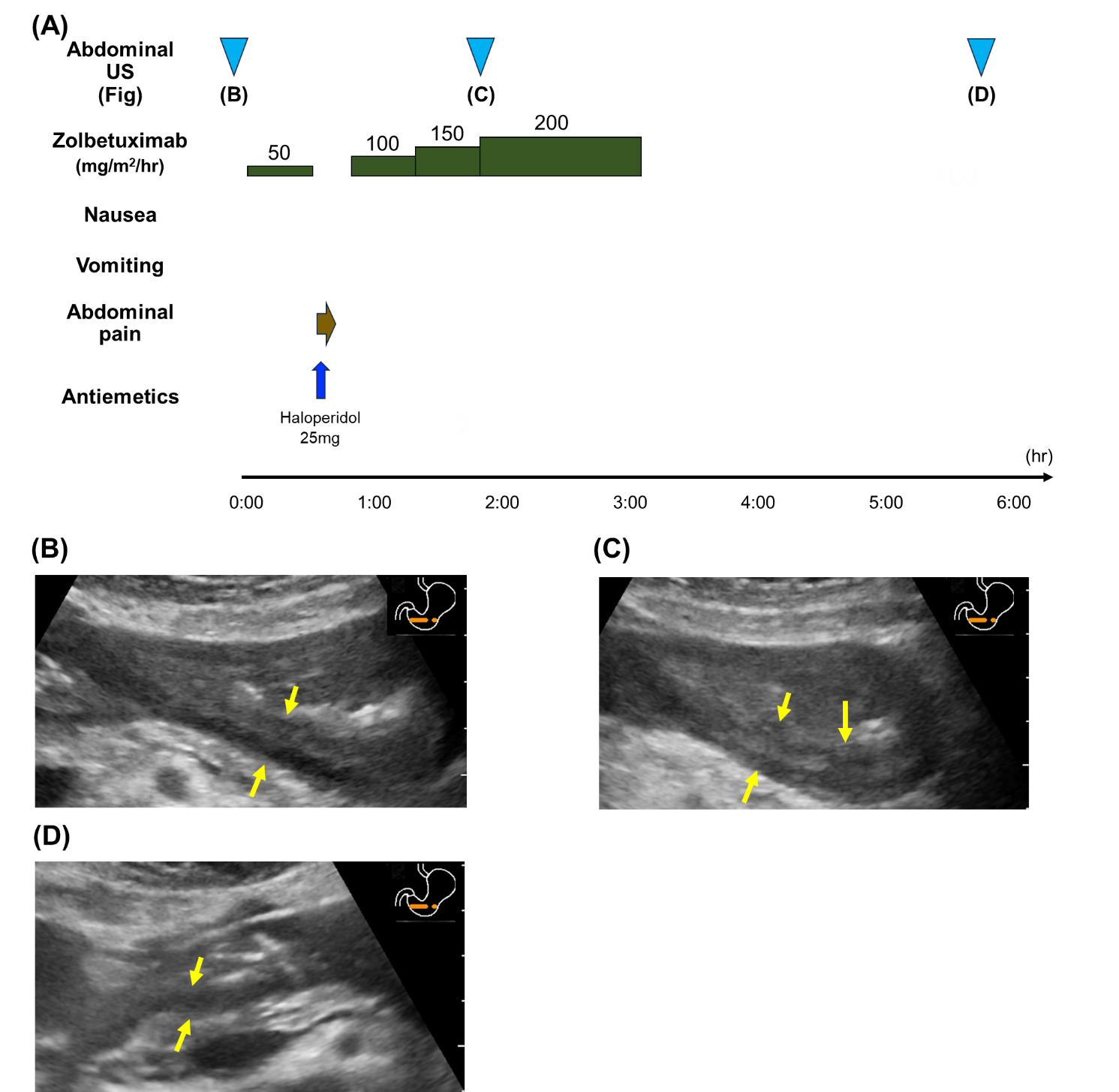
Supplementary Figures

**Supplementary Figure 1.** (**A**) Treatment course with the second cycle of zolbetuximab in Case 2.(**B**) At baseline, ultrasonography showed a reduction in gastric wall thickening compared with that observed during the first treatment cycle. **(C**) A slightly increased echo intensity of the submucosal layer was observed during zolbetuximab. (**D**) Increased echogenicity in the submucosa disappeared after the completion of zolbetuximab administration


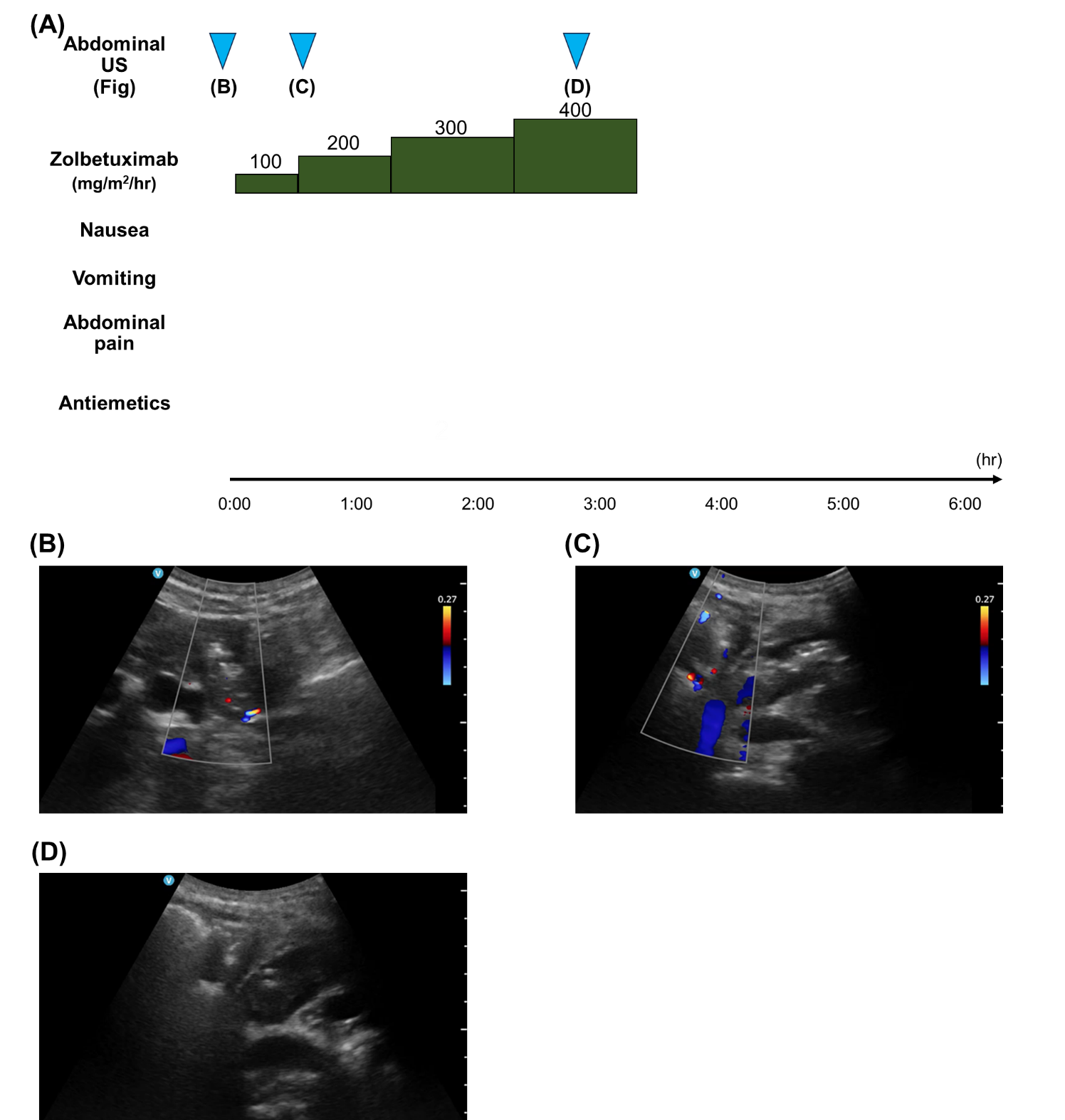


**Supplementary Figure 2** **(A)**Treatment course with the first cycle of zolbetuximab in Case 3. US imaging **(B)** at baseline, **(C)** during zolbetuximab administration **(D)** after completion of zolbetuximab administration. No significant US changes were observed in the examined small intestine.

**Supplementary Table 1.** **Summary of US findings**


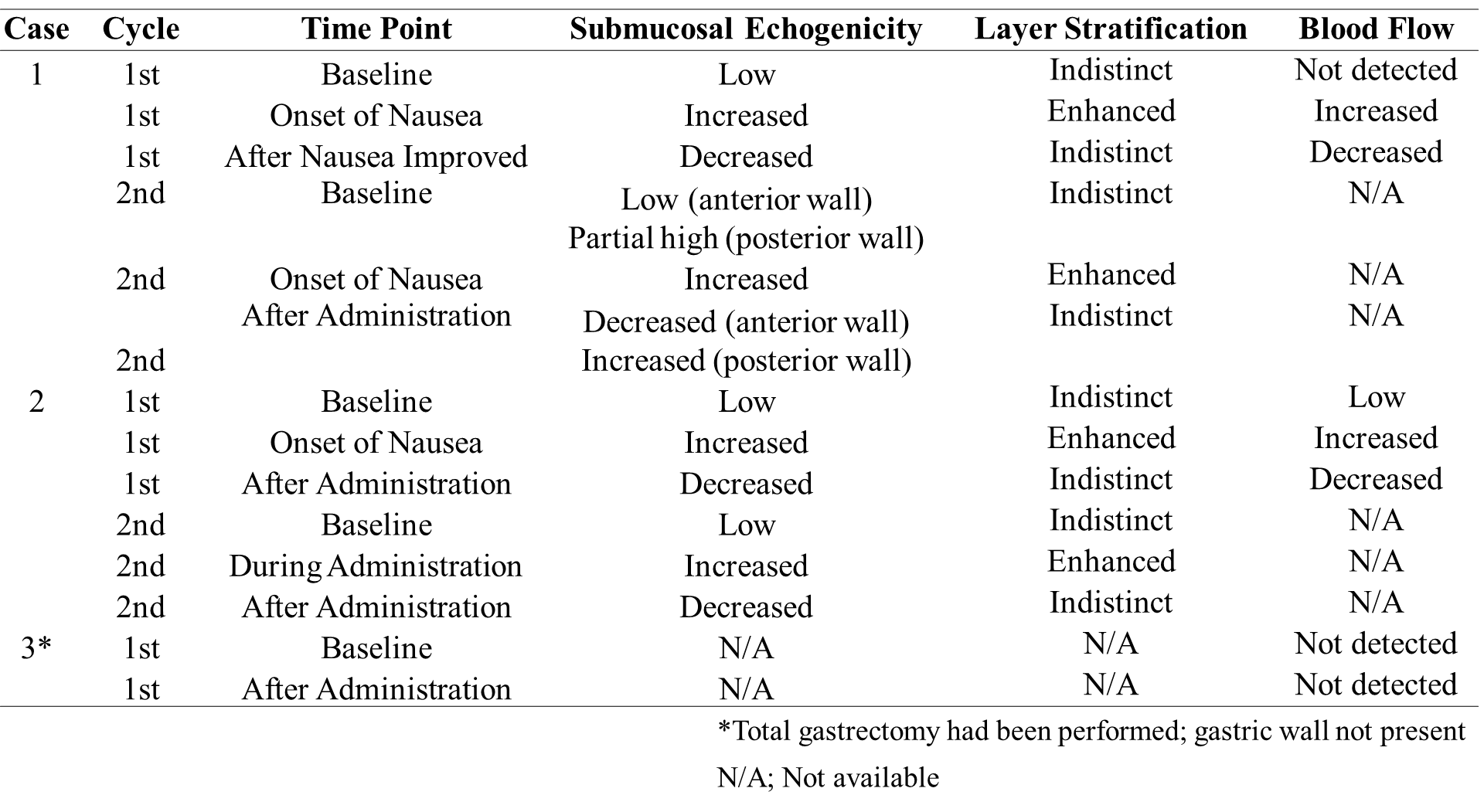

Supplement: Supplementary file 1 [file DataSheet1.docx]
